# Unmasking deep-rooted trauma: long-term effects of childhood adversities on posttraumatic stress disorder in healthcare workers facing acute multi-trauma

**DOI:** 10.3389/fpsyt.2026.1788332

**Published:** 2026-04-14

**Authors:** Elie G. Karam, Josleen Al Barathie, Jana Mansour Jamaleddin, Katherine Keyes

**Affiliations:** 1 Institute for Development, Research, Advocacy, and Applied Care (IDRAAC), Beirut, Lebanon; 2 Department of Psychiatry and Clinical Psychology, Saint George University of Beirut Faculty of Medicine, Beirut, Lebanon; 3 Department of Psychiatry and Clinical Psychology, Saint George Hospital University Medical Center, Beirut, Lebanon; 4 Department of Epidemiology, Mailman School of Public Health, Columbia University, New York, NY, United States

**Keywords:** adverse childhood experiences (ACEs), Beirut port blast, economic crisis, healthcare workers, posttraumatic stress disorder (PTSD), subthreshold PTSD

## Abstract

**Purpose:**

In recent years, healthcare workers (HCWs) in Lebanon have encountered compounded traumatic exposures, including the Beirut Port blast, COVID-19, and an ongoing economic crisis, often preceded by early-life adversities such as adverse childhood experiences (ACEs). Understanding how these acute stressors interact with early adversities is crucial for assessing their long-term psychological impact. Accordingly, this study examines the extent to which these combined factors predict the development of full and subthreshold posttraumatic stress disorder (PTSD) over time.

**Methods:**

A cohort study was conducted following 296 HCWs from Saint George Hospital University Medical Center, with assessments at two timepoints: 6–7 months and 2–2.5 years after the Beirut Blast. PTSD symptoms were measured using the PCL-5, applying both full-threshold criteria and six definitions of subthreshold PTSD. Bivariable and multivariable analysis were conducted.

**Results:**

At 6–7 months, acute stressors (financial hardship, Beirut Blast, and COVID-19) were significantly associated with PTSD across most definitions. However, by 2–2.5 years, ACEs became the strongest and most consistent predictor of both full-threshold and subthreshold PTSD, while the impact of acute stressors diminished.

**Conclusion:**

The impact of acute trauma on the risk of PTSD fades over time, while early-life adversity has an enduring impact. The findings highlight the importance of including developmental trauma histories in PTSD assessments. In concordance with stress sensitization and neurobiological models, the results indicate a marked temporal shift, where the diminishing effects of acute stressors give way to the enduring role of early life adversity in shaping PTSD symptom trajectories.

## 1 Introduction

Posttraumatic stress disorder (PTSD) is a severe psychiatric condition that emerges following exposure to a traumatic event, leading to substantial distress and impairment in daily functioning. Diagnoses of PTSD are characterized by exposure to a traumatic event (Criterion A) (1–3) and the presence of at least six out of twenty symptoms across four symptom clusters: intrusion (Cluster B), avoidance (Cluster C), alterations in mood and cognition (Cluster D), and hyper-arousal and reactivity (Cluster E) (1). These symptoms must persist for over a month and significantly disrupt an individual’s psychological well-being. While this classification provides a structured framework for diagnosing PTSD, research has increasingly recognized the presence of substantial posttraumatic symptomatology in individuals who do not meet the full diagnostic criteria but still experience considerable impairment, referred to as subthreshold PTSD.

Subthreshold PTSD, also described as partial or subsyndromal PTSD, individuals who exhibit PTSD-related distress but do not fulfill the DSM-defined criteria. Although not an officially recognized diagnosis, subthreshold PTSD is associated with negative health outcomes, increased psychiatric comorbidities, and functional impairment comparable to full-threshold PTSD (2–4). However, defining subthreshold PTSD remains a challenge due to the variability in classification criteria across studies. Franklin et al. identified four primary definitions: the Five-Symptom definition (requiring at least one symptom from each DSM-5 cluster), “Definition-1” (where individuals meet Criteria B, C, and D or Criteria B, C, and E), the “Six-Plus” definition (requiring six PTSD symptoms without full diagnostic criterion fulfillment), and the “Majority” definition (meeting full criteria for three out of the four symptom clusters) (5). Similarly, McLaughlin et al. proposed two alternative definitions based on symptom distribution: the “2-Cluster” definition (meeting full-threshold criteria for two clusters) and the “1-Cluster” definition (meeting criteria for only one cluster) (6). Understanding both full-threshold and subthreshold PTSD is essential for early intervention and targeted support of at-risk populations.

Economic hardship—before or after trauma—is a strong predictor of PTSD symptomatology. The experience or anticipation of financial insecurity following trauma exposure has been linked to heightened PTSD symptoms (7). In Greece, a study of 1,208 lower middle-class residents found that 59.6% scored above the clinical threshold for PTSD symptoms after drastic salary cuts, business closures, and job losses (8). Similarly, a Canadian study of women in Fort McMurray found that COVID-19–related job loss significantly increased PTSD risk fivefold (9). Studies in war-exposed populations also highlight financial strain as a risk factor; among Iraq and Afghanistan veterans, those screening positive for PTSD were more likely to lack resources for basic expenses (10). In Lebanon, where economic instability is pervasive, research after the Beirut Blast found that a higher crowding index and lower financial well-being significantly predicted greater PTSD prevalence (11). These findings align with broader research suggesting that individuals with lower income and greater fear of financial instability exhibit higher PTSD symptoms (12–14). Despite extensive evidence on financial crises predicting full-threshold PTSD, their longitudinal role in predicting symptoms across full- and sub-threshold definitions in poly-trauma–exposed populations remain underexamined.

An emerging research focus is the relationship between COVID-19 and PTSD. The pandemic has been conceptualized as a protracted traumatic event, characterized by prolonged uncertainty, social isolation, and loss (15). A meta-analysis estimated the pooled PTSD prevalence related to COVID-19 at 22.6% (16), comparable to rates observed following natural disasters and terrorist attacks (17, 18). Healthcare workers (HCWs) have been particularly affected, with a systematic review revealing a 34% pooled incidence of PTSD symptoms among HCWs during the pandemic (19). Factors such as occupational role, increased level of exposure, fewer years of experience, female gender, and being unmarried were identified as key contributors to PTSD risk (20, 21). Research has also examined subthreshold PTSD among COVID-19 survivors and HCWs, showing strong links between COVID-related stressors and subthreshold symptoms (22, 23). However, as most studies on COVID-19’s predictive role are cross-sectional, it remains crucial to assess whether its impact persists years later, especially alongside more salient traumatic events.

Beyond COVID-19 and economic stressors, childhood adversity is also a well-established predictor of PTSD. Adverse Childhood Experiences (ACEs), including abuse, neglect, and parental loss, have been consistently associated with heightened vulnerability to PTSD across diverse populations (24–26). Studies have demonstrated a dose-response relationship between the number of ACEs and PTSD severity, with individuals exposed to four or more ACEs exhibiting significantly higher PTSD symptomatology (27). In war-exposed populations, such as Syrian refugee children in Lebanon, ACEs have been shown to independently predict PTSD beyond the effects of war exposure (28).

Further evidence highlights ACEs as a major contributor to early-onset psychiatric disorders, with nearly one-third of adolescent cases linked to these adversities, particularly PTSD (29). Among African American youth survivors of urban violence in the US, a positive relationship between ACEs and PTSD symptomatology was established, further validating the well-established increased vulnerability (30, 31). A nationwide Danish cohort study also corroborates this link, demonstrating a graded association between ACE trajectories and PTSD diagnoses in young adulthood, with the highest risk observed in individuals experiencing multiple, severe adversities (32). From thereon, the aforementioned indicates an established significant link between ACEs’ role in predicting full-threshold PTSD and a lesser explored but significant association between x and sub-threshold PTSD prediction. However, evidence on the predictive significance of ACEs across varying timepoints following trauma exposure, various subthreshold conceptualizations of PTSD, and in the context of polytraumatized is scarce at best.

Recent research links human-made disasters (war, terrorism, industrial accidents) to higher PTSD prevalence than natural disasters (33, 34). A study (35) across 18 World Mental Health Surveys found PTSD rates 26.6% higher after human-made crises, with greater odds compared to natural disaster exposure.

Following the Beirut Port Blast, Maalouf et al. (36) reported that 51.5% of 1,517 Lebanese children aged 8–17 years met criteria for probable PTSD, with proximity to the blast, witnessing casualties, and sustaining injuries emerging as significant predictors. Similarly, among 419 Lebanese university students, 26% met probable PTSD criteria, with the Beirut Blast identified as the traumatic event (11). In a separate study of 519 Lebanese HCWs, 44% were at risk for PTSD symptoms four months post-blast, with significant predictors including physical injury, COVID-19 diagnosis, number of injuries sustained, and loss of a loved one (OR = 4.23) (13).

Given the above, this study aims to predict subthreshold and full PTSD among HCWs in Lebanon by examining the impact of multiple stressors, including the Beirut Port Blast, the COVID-19 pandemic, childhood adversities, and the ongoing financial crisis. The August 4, 2020 Beirut Port Blast, the largest non-nuclear explosion in modern history, had caused over 200 deaths, 6,000 injuries, and displaced 300,000 people. The economic crisis exacerbated stressors, with the Lebanese currency losing 98% of its value by 2022, causing widespread hardship. The COVID-19 pandemic further strained healthcare, exposing HCWs to sustained physical and psychological stress.

By integrating these interconnected stressors into our predictive model, we aimed to assess the relative contribution of acute versus distal stressors over time, using longitudinal data collected post-blast, on PTSD risk among HCWs, acknowledging the quadruple effect of those exposures together.

## 2 Materials and methods

### 2.1 Study design and setting

This longitudinal cohort study was conducted in Beirut after the Port explosion. Data were collected in four waves: 9–15 days, 21–27 days, 6–7 months, and 2–2.5 years post-explosion. The current analysis uses waves 3 and 4, as waves 1 and 2 fall within the Acute Stress Disorder timeframe, while our focus is on PTSD.

### 2.2 Participants and recruitment

The study included HCWs from Saint George Hospital University Medical Center (SGHUMC) in Beirut. All staff aged 18 or older—clinical, administrative, and support—were eligible, with no exclusions. The first wave of data collection happened in person using self-filled questionnaires and 570 participants managed to complete the survey after consenting orally. Later waves of data collection took place online by disseminating survey links through email, SMS, WhatsApp, and QR-coded letters to the hospital’s employees. Although the main focus of the study was to follow-up participants across the four waves, we also recruited new participants in each interval to maximize our sample size. Therefore, the final samples of waves 2, 3, and 4 were 733, 808, and 524 respectively with response rates of 38%, 41.88% and 40.5%, respectively. The final sample comprised 296 participants followed across the two waves (3 & 4) analyzed here.

## 3 Measures

### 3.1 Sociodemographic characteristics

Participants provided information on their age (in years), gender, and professional role (clinical or non-clinical).

### 3.2 Beirut port blast exposure

Exposure was measured with the Beirut Port Blast Exposure Inventory, capturing nine types: (1) location during explosion, (2) personal injuries, (3) difficulty accessing care, (4) mild injury to loved one, (5) severe injury to loved one, (6) death of loved one, (7) home damage, (8) rescue participation, (9) witnessing mutilated/deceased individuals. The Beirut Port Blast Exposure Inventory is a checklist which was developed based on the experience and input of local experts familiar with the event.

A weighted exposure score reflected the cumulative severity of exposure events. A panel of 20 departmental experts (clinicians, statisticians, and public health professionals) independently rated each exposure type from 0 to 100, with higher scores indicating greater severity. Final weights were the median of expert ratings, and each participant’s cumulative score was the sum of weights for all exposures experienced. **Supplementary Table** (**Supplementary Table 1**) presents the final weights.

### 3.3 Previous trauma exposure and reaction

Participants reported prior trauma, including major accidents, serious illness, loss of a loved one (unrelated to the explosion), and past exposure to conflict. Childhood trauma (neglect, physical, or sexual abuse) was also assessed using three questions presented in **Supplementary Table 2**. All responses were binary (Yes/No), and separate count scores were created for childhood and personal trauma.

### 3.4 Economic situation

Shifts in financial and lifestyle conditions over the past year were assessed. Participants reported changes in their ability to meet financial needs and in essential aspects of daily life (children’s education, healthcare access, grocery shopping), as well as leisure activities (dining out, traveling). They also indicated whether the number of household income contributors had changed. A financial hardship score was calculated by summing reported changes.

### 3.5 COVID-19 exposure score

A composite score was developed to assess the impact of COVID-19-related stressors. Items included: violence due to being a HCW, proximity to COVID patients, PPE provision, loss of loved ones, isolation days, death of cared-for patients, stigmatization/discrimination, patient triage, fear of infection, fear of infecting loved ones, trust in workplace, trust in government, and training for transmission prevention. The COVID-19 questions were taken from the international HEROES cohort study. These questions were designed to comprehensively capture relevant acute exposures that could be experienced by healthcare workers. To assign weights, a process similar to the Beirut Port Blast was used: experts independently rated each item’s impact on a 0–5 scale, and the final weight was the median rating. A **Supplementary Table** (**Supplementary Table 3**) lists the items and their assigned weights.

### 3.6 Depression

Depressive symptoms were assessed using the 9-item Patient Health Questionnaire (PHQ-9). Items were rated from 0 (“Not at all”) to 3 (“Nearly every day”), yielding total scores of 0–27. A cut-off of ≥10 indicated clinically significant symptoms, with sensitivity of 0.85 and specificity of 0.89 (37).

### 3.7 PTSD

PTSD symptoms were assessed using the 20-item PTSD Checklist for DSM-5 (PCL-5). Symptoms were rated on a 5-point scale from “Not at all” (0) to “Extremely” (4). Full-threshold PTSD was determined based on DSM-5 criteria, requiring endorsement of at least six symptoms, including at least one symptom from Criterion B (intrusion), one from Criterion C (avoidance), two from Criterion D (negative alterations in mood/cognition), and two from Criterion E (hyperarousal).

Alongside full PTSD, several subthreshold definitions were applied to capture varying degrees of symptom severity:

- Five Symptoms: At least one symptom endorsed from each PTSD criterion.
- Definition-1: Meeting the criteria for B, C, and D or B, C, and E.
- Six Plus: Presence of at least six PTSD symptoms, regardless of their distribution across criteria.
- Majority: Meeting full-threshold PTSD criteria for at least three out of the four symptom clusters (B-E).
- 2-Clusters: Meeting full-threshold PTSD criteria for at least two out of the four symptom clusters (B-E).
- 1-Cluster: Meeting full-threshold PTSD criteria for at least one of the four symptom clusters (B-E).

### 3.8 Ethical considerations

The Institutional Review Board of SGHUMC Faculty of Medicine, University of Balamand, approved the study, which is registered with the U.S. Office of Human Research Protections (OHRP). Due to its longitudinal nature, data were not anonymized but securely stored on a password-protected computer accessible only to the research team. Electronic informed consent was obtained before participation.

### 3.9 Statistical analysis

Analyses were conducted in Stata v.17. Descriptive statistics used means (SD) for continuous variables and frequencies (%) for categorical variables. Bivariate associations were assessed using chi-square (χ²) tests for categorical variables and independent-samples t-tests for continuous variables. Also, bivariate logistic regression tested associations with PTSD outcomes (full and subthreshold). Multivariable logistic regression models, run separately by wave, adjusted for gender, age, profession, depression, blast exposure, financial and COVID-19 scores, prior trauma, and childhood adversity. The primary output from these logistic models is the Odds Ratio (OR), which quantifies the magnitude and direction of the association. An OR greater than 1 indicates higher odds of the outcome for that category relative to the reference group, whereas an OR less than 1 indicates lower odds. Missing data were addressed via complete case analysis. Specifically, participants were included in the analyses only if they had available data on the variables required for the longitudinal analyses across the relevant study waves. Individuals with missing information on the key variables or without follow-up data linking them to previous waves were excluded from the analytic sample.

## 4 Results

### 4.1 Descriptive

Most participants were female (78.6%).

At 6–7 months post-blast, the mean age of participants was 40.6 ± SD: 12.17 years. The majority worked in clinical roles (69.9%). Mental health burden was high: 29.1% met full PTSD criteria, 12.6% had depression, and many had subthreshold PTSD (five symptoms: 36.1%; six-symptom: 50.4%; two-cluster: 60.4%). Prior adversities were common (mean prior trauma = 1.33 ± 0.99), with 22.57% reporting any of the three childhood adversity types. Financial hardship (mean = 2.65 ± 1.23), COVID-19 impact (mean = 18.29 ± 7.31), and Beirut Blast exposure (mean = 177.37 ± 127.75) were also prominent (**Table 1**).

**Table 1:** Descriptive characteristics of the sample at 6–7 months and 2–2.5 years post-Beirut port blast.

Variables		6–7 Months		2–2.5 Years	
		N	%	N	%
Gender	*Female*	231	78.57	231	78.57
	*Male*	63	21.43	63	21.43
Age^		40.6 ± 12.17		43.8 ± 12.13	
Profession	*Non clinical*	89	30.07	64	21.62
	*Clinical*	207	69.93	232	78.38
Depression	*No*	230	87.45	233	86.62
	*Yes*	33	12.55	36	13.38
PTSD	*No*	210	70.95	264	89.19
	*Yes*	86	29.05	32	10.81
Five Symptoms	*No*	184	63.89	210	80.46
	*Yes*	104	36.11	51	19.54
Definition 1	*No*	177	61.46	208	79.69
	*Yes*	111	38.54	53	20.31
Two Cluster	*No*	114	39.58	120	45.98
	*Yes*	174	60.42	141	54.02
One Cluster	*No*	54	18.75	65	24.9
	*Yes*	234	81.25	196	75.1
Majority	*No*	152	52.78	190	72.8
	*Yes*	136	47.22	71	27.2
Six Symptoms	*No*	143	49.65	154	59.23
	*Yes*	145	50.35	106	40.77
Beirut Port Blast^		177.37 ± 127.75		181.53 ± 124.9	
Financial Score^		2.65 ± 1.23		2.52 ± 1.24	
Covid-19 Score^		18.29 ± 7.31		15.31 ± 6.57	
Prior Trauma Score^		1.33 ± 0.99		1.32 ± 0.97	
Childhood Adversities	No CA	199	77.43	189	74.41
	Any CAs	58	22.57	65	25.59

^^^Mean and SD for continuous variables.

At 2–2.5 years post-blast, clinical professionals comprised 78.38% (n = 232). PTSD prevalence declined modestly to 10.81% (n = 32), while depression rates were similar to the previous wave (13.38%, n = 36). PTSD symptom distribution shifted, with fewer participants showing extensive subthreshold cluster involvement (19.54% reported five symptoms; 40.77% met the six-symptom threshold; 54.02% met the two-cluster definition). Financial hardship eased slightly (mean = 2.52 ± 1.24), and COVID-19 burden dropped markedly (mean = 15.31 ± 6.57), reflecting broader recovery trends. Prior trauma exposure remained stable (mean = 1.32 ± 0.97), and the Beirut Port Blast’s impact persisted as a significant contextual factor (mean = 181.53 ± 124.9) (**Table 1**).

### 4.2 Bivariate

6–7 months post-blast, bivariate analysis showed significant PTSD links with demographic/psychosocial factors (**Table 2**; effect size in **Table 3**). Females had higher PTSD rates across all definitions (full: 32.47% vs 17.46%, p = 0.02 and OR = 2.38, p = 0.015; 5 symptoms: 40.89% vs 19.67%, p = 0.002 and OR = 2.94, p = 0.002; definition 1: 44.44% vs 18.03%, p < 0.001 and OR = 3.70, p <0.001; two-cluster: 64.44% vs 45.9%, p = 0.03 and OR = 2.04, p = 0.011; six-symptom: 54.67% vs 36.01%, p = 0.01 and OR = 2.27, p = 0.004). Depression emerged as a strong correlate, with those meeting depression criteria consistently demonstrating an increased likelihood of PTSD in all definitions as well (e.g., six-symptom threshold: 87.88% vs. 12.12%, p < 0.001 and OR = 8.63, p < 0.001; two-cluster: 84.85% vs. 15.15%, p = 0.003 and OR = 4.08, p = 0.005; definition 1: 60.61% vs. 39.39%, p = 0.005 and OR = 2.83, p = 0.006; five-symptom: 57.58% vs. 42.42%, p = 0.006 and OR = 2.75, p = 0.008; full PTSD: 51.52% vs. 48.48%, p = 0.004 and OR = 2.88, p = 0.005). Severity of Beirut Blast exposure, financial hardship, COVID-19 burden, and prior trauma scores were higher among individuals with PTSD across all classifications (Blast: 214.78 vs 161.83, p = 0.001 and OR = 1.003, p = 0.001; Financial: 3.27 vs 2.41, p < 0.001 and OR = 2.00, p < 0.001; COVID-19: 20.05 vs 17.58, p = 0.01, OR = 1.05, p = 0.011; Prior Trauma: 1.7 vs 1.22, p = 0.007 and OR = 1.61, p = 0.009 for full PTSD). Childhood adversities did not consistently predict PTSD (e.g., p = 0.953, and OR = 0.95, p = 0.875 for full PTSD).

**Table 2:** Bivariable analysis of PTSD across six definitions, 6–7 months post-Beirut blast.

6–7 months post-blast	No PTSD (full)	Yes PTSD (full)	P-value	No PTSD (5 symptom)	Yes PTSD (5 symptom)	P-value	No PTSD (definition 1)	Yes PTSD (definition 1)	P-value	No PTSD (2 cluster)	Yes PTSD (2 cluster	P-value	No PTSD (majority)	Yes PTSD (majority)	P-value	No PTSD (6 symptom)	Yes PTSD (6 symptom)	P-value
	N (%)	N (%)		N (%)	N (%)		N (%)	N (%)		N (%)	N (%)		N (%)	N (%)		N (%)	N (%)	
Gender																		
*Female*	156 (67.53)	75 (32.47)	0.02*	133 (59.11)	92 (40.89)	0.002*	125 (55.56)	100 (44.44)	<0.001*	80 (35.56)	145 (64.44)	0.03*	112 (49.78)	113 (50.22)	0.05	102 (45.33)	123 (54.67)	0.01*
*Male*	52 (82.54)	11 (17.46)		49 (80.33)	12 (19.67)		50 (81.97)	11 (18.03)		33 (54.1)	28 (45.9)		39 (63.93)	22 (36.07)		39 (63.93)	22 (36.07)	
Age^	39.96 ± 12.0	42.18 ± 12.5	0.167	40.28 ± 12	41.5 ± 12.59	0.4264	40.18 ± 12.1	41.57 ± 12.39	0.3541	40.59 ± 12.43	40.8 ± 12.1	0.885	40.68 ± 12.22	40.76 ± 12.24	0.995	40.4 ± 12	41.04 ± 12.45	0.6632
Profession																		
*Non clinical*	62 (69.66%)	27 (30.34)	0.75	57 (64.04)	32 (35.96)	0.971	56 (62.92)	33 (37.08)	0.733	38 (42.7)	51 (57.3)	0.47	50 (32.89)	102 (67.11)	0.439	51 (57.3)	38 (42.7)	0.082
*Clinical*	148 (71.5%)	59 (28.5)		127 (63.82)	72 (36.18)		121 (60.8)	78 (39.2)		76 (38.19)	123 (61.81)		39 (28.68)	97 (71.32)		92 (46.23)	107 (53.77)	
Depression																		
*No*	168 (73.04%)	62 (26.96)	0.004*	154 (66.96)	76 (33.04)	0.006*	149 (64.78)	81 (35.22)	0.005*	97 (42.17)	133 (57.83)	0.003*	130 (94.2)	8 (5.8)	0.001*	125 (54.35)	105 (45.65)	<0.001*
*Yes*	16 (48.48%)	17 (51.52)		14 (42.42)	19 (57.58)		13 (39.39)	20 (60.61)		5 (15.15)	28 (84.85)		100 (80)	25 (20)		4 (12.12)	29 (87.88)	
Beirut Port Blast^	161.83 ± 117.92	214.78 ± 142.69	0.001*	158.88 ± 116.41	209.05 ± 142.21	0.0013*	160.33 ± 117.14	203.56 ± 141.38	0.0052*	145.67 ± 109.22	197.52 ± 135.95	0.0007*	151.16 ± 113.67	205.87 ± 137.83	0.0003*	151.31 ± 112.74	202.32 ± 137.9	0.0007*
Financial Score^	2.41 ± 1.27	3.27 ± 0.89	<0.001*	2.38 ± 1.27	3.15 ± 0.98	<0.001*	2.35 ± 1.27	3.14 ± 0.99	<0.001*	2.16 ± 1.31	2.98 ± 1.05	<0.001*	2.23 ± 1.26	3.13 ± 1	<0.001*	2.19 ± 1.29	3.11 ± 0.96	<0.001*
Covid-19 Score^	17.58 ± 6.77	20.05 ± 8.26	0.01*	17.25 ± 6.64	20.19 ± 8.07	0.0012*	16.94 ± 6.55	20.5 ± 7.94	0.0001*	16.64 ± 6.73	19.38 ± 7.48	0.002*	16.76 ± 6.62	20.03 ± 7.67	0.0002*	16.63 ± 6.54	19.95 ± 7.66	0.0001*
Prior Trauma Score^	1.22 ± 0.97	1.7 ± 1.02	0.007*	1.17 ± 0.94	1.71 ± 1.04	0.0009*	1.17 ± 0.97	1.68 ± 0.99	0.0013*	1.01 ± 0.92	1.62 ± 0.99	<0.001*	1.13 ± 0.95	1.62 ± 1.01	0.0006*	1.14 ± 0.96	1.59 ± 1.02	0.0024*
Childhood Adversities																		
*No CA*	142 (71.36)	57 (28.64)	0.953	130 (65.33)	69 (34.67)	0.696	125 (62.81)	74 (37.19)	0.945	86 (43.22)	113 (56.78)	0.046*	112 (56.28)	87 (43.72)	0.343	105 (52.76)	94 (47.24)	0.203
*Any CA*	42 (72.41)	16 (27.57)		36 (63.16)	21 (36.84)		35 (60.34)	23 (39.66)		18 (31.03)	40 (68.97)		28 (48.28)	30 (51.72)		25 (43.1)	33 (56.9)	

^*p-value < 0.05.^

^^^t-test for continuous variables.

**Table 3:** Simple logistic regression of PTSD by definition type, 6–7 months post-Beirut port blast.

6–7 months post-blast	PTSD (full)	P-value	PTSD (5 symptom)	P-value	PTSD (definition 1)	P-value	PTSD (2 cluster)	P-value	PTSD (majority)	P-value	PTSD (6 symptoms)	P-value
Gender												
*Female*	Ref	0.015*	Ref	0.002*	Ref		Ref		Ref		Ref	
*Male*	0.42		0.34		0.27	<0.001*	0.49	0.011*	0.60	0.071	0.44	0.004*
Age^	1.01	0.167	1.008*	0.425	1.009	0.353	1.001	0.884	1.001	0.955	1.004	0.662
Profession												
*Non clinical*	Ref	0.750	Ref	0.971	Ref		Ref	0.470	Ref		Ref	
*Clinical*	0.92		1.009		1.09	0.733	1.21		1.22	0.440	1.56	0.083
Depression												
*No*	Ref	0.005*	Ref	0.008*	Ref		Ref		Ref		Ref	
*Yes*	2.88		2.75		2.83	0.006*	4.08	0.005*	4.06	0.001*	8.63	<0.001*
Beirut Port Blast^	1.003	0.001*	1.003	0.002*	1.003	0.006*	1.01	0.001*	1.003	<0.001*	1.003	0.001*
Financial Score^	2.00	<0.001*	1.85	<0.001*	1.86	<0.001*	1.78	<0.001*	2.01	<0.001*	2.03	<0.001*
Covid-19 Score^	1.05	0.011*	1.06	0.002*	1.07	<0.001*	1.06	0.002*	1.07	<0.001*	1.07	<0.001*
Prior Trauma Score^	1.61	0.009*	1.74	0.001*	1.69	0.002*	1.98	<0.001*	1.72	0.001*	1.61	0.003*
Childhood Adversities												
*No CA*	Ref	0.875	Ref	0.829	Ref	0.733	Ref		Ref		Ref	
*Any CA*	0.95		1.07		1.11		1.69	0.098	1.38	0.282	1.48	0.197

*p-value < 0.05.

^^^continuous variables.

At 2–2.5 years post-blast, PTSD was significantly linked to depression, prior trauma, and childhood adversity—across all definitions—with higher rates among those affected (e.g., for PTSD six-symptom: depression 90.32% vs. 33.02%, *p* < 0.001, and OR = 18.93, p <0.001*, and prior trauma 1.87 vs. 0.97, *p* < 0.001, OR = 3.24, p < 0.001; for PTSD full: childhood adversity 20.00% vs. 3.17%, *p* < 0.001, and OR = 7.63, p < 0.001) (**Table 4** and effect size in **Table 5**). Exposure to the Beirut Port Blast was also consistently associated with PTSD, with affected individuals showing significantly higher PTSD rates (e.g., full PTSD: 251.0 vs. 172.9, *p* = 0.0008 and OR = 1.004, p = 0.001). In contrast, gender, profession, financial score, and COVID-19 impact were not significantly associated with PTSD across definitions. Age showed a marginal association in one definition (PTSD majority: 41.49 vs 44.94 ± 12.21, *p* = 0.0419, and OR 0.98, p = 0.043) but lacked consistent significance.

**Table 4:** Bivariable analysis of PTSD across six definitions, 2-2.5 years post-Beirut blast.

2-2.5 years post-blast	No PTSD (full)	Yes PTSD (full)	P-value	No PTSD (5 symptom)	Yes PTSD (5 symptom)	P-value	No PTSD (definition 1)	Yes PTSD (definition 1)	P-value	No PTSD (2 cluster)	Yes PTSD (2 cluster	P-value	No PTSD (majority)	Yes PTSD (majority)	P-value	No PTSD (6 symptom)	Yes PTSD (6 symptom)	P-value
	N (%)	N (%)		N (%)	N (%)		N (%)	N (%)		N (%)	N (%)		N (%)	N (%)		N (%)	N (%)	
Gender																		
*Female*	205 (88.74)	26 (11.26)	0.69	160 (78.82)	43 (21.18)	0.251	160 (78.82)	43 (21.18)	0.585	93 (45.81)	110 (54.19)	0.935	145 (71.43)	58 (28.57)	0.426	122 (60.4)	80 (39.6)	0.358
*Male*	57 (90.48)	6 (9.52)		48 (85.71)	8 (14.29)		46 (82.14)	10 (17.86)		26 (46.43)	30 (53.57)		43 (76.79)	13 (23.21)		30 (53.57)	26 (46.43)	
Age^	43.92 ± 12	42.91 ± 13.28	0.656	44.51 ± 11.92	41.92 ± 13.16	0.175	44.69 ± 12.15	41.3 ± 12.09	0.07	44.4 ± 11.96	43.66 ± 12.42	0.625	44.94 ± 12.21	41.49 ± 11.87	0.0419*	43.99 ± 11.67	43.92 ± 12.98	0.9596
Profession																		
Non clinical	55 (85.94)	9 (14.06)	0.34	49 (81.67)	11 (18.33)	0.788	47 (78.33)	13 (21.67)	0.765	30 (50)	30 (50)	0.476	41 (68.33)	19 (31.67)	0.376	35 (58.33)	25 (41.67)	0.872
Clinical	209 (90.09)	23 (9.91)		161 (80.1)	40 (19.9)		161 (80.1)	40 (19.9)		90 (44.78)	111 (55.22)		149 (74.13)	52 (25.87)		119 (59.5)	81 (40.5)	
Depression																		
*No*	216 (92.7)	17 (7.3)	<0.001*	179 (84.43)	33 (15.57)	<0.001*	178 (83.96)	34 (16.04)	<0.001*	111 (52.36)	101 (47.64)	<0.001*	166 (78.3)	46 (21.7)	<0.001*	142 (66.98)	70 (33.02)	<0.001*
*Yes*	26 (72.22)	10 (27.78)		18 (58.06)	13 (41.94)		17 (54.84)	14 (45.16)		4 (12.9)	27 (87.1)		11 (35.48)	20 (64.52)		3 (9.68)	28 (90.32)	
Beirut Port Blast^	172.91 ± 125.18	251 ± 99.62	0.0008*	167.66 ± 128.52	235.91 ± 90.05	0.0004*	169.93 ± 128.15	224.51 ± 100.3	0.0043*	145.87 ± 126.01	211.51 ± 115.73	<0.001*	164.06 ± 126.56	226.22 ± 108.31	0.0003*	152.95 ± 124.14	221.64 ± 115.05	<0.001*
Financial Score^	2.52 ± 1.22	2.5 ± 1.37	0.93	2.56 ± 1.21	2.42 ± 1.34	0.484	2.55 ± 1.22	2.46 ± 1.28	0.65	2.53 ± 1.26	2.53 ± 1.21	0.997	2.54 ± 1.22	2.5 ± 1.28	0.815	2.54 ± 1.22	2.5 ± 1.26	0.807
Covid-19 Score^	15.21 ± 6.54	16.09 ± 6.82	0.47	15.4 ± 6.65	15.17 ± 6.2	0.829	15.31 ± 6.54	15.53 ± 6.63	0.829	15.72 ± 6.56	15.04 ± 6.55	0.405	15.53 ± 6.54	14.87 ± 6.62	0.469	15.54 ± 6.52	15.14 ± 6.64	0.635
Prior Trauma Score^	1.25 ± 0.96	2.04 ± 0.86	0.0001*	1.23 ± 0.94	1.79 ± 1.01	0.0005*	1.25 ± 0.97	1.67 ± 0.93	0.009*	1.02 ± 0.77	1.61 ± 1.06	<0.001*	1.18 ± 0.95	1.76 ± 0.94	<0.001*	0.97 ± 0.74	1.87 ± 1.04	<0.001*
Childhood Adversities																		
*No CA*	183 (96.83)	6 (3.17)	<0.001*	154 (91.12)	15 (8.88)	<0.001*	151 (89.35)	18 (10.65)	<0.001*	97 (57.4)	72 (42.6)	<0.001*	144 (85.21)	25 (14.79)	<0.001*	125 (73.96)	44 (26.04)	<0.001*
*Any CA*	52 (80)	13 (20)		38 (63.33)	22 (36.67)		39 (65)	21 (35)		17 (28.81)	42 (71.19)		35 (58.33)	25 (41.67)		23 (38.98)	36 (61.02)	

*p-value < 0.05.

^^^t-test for continuous variables.

**Table 5:** Simple logistic regression of PTSD by definition type, 2-2.5 years post-Beirut port blast.

2-2.5 years post-blast	PTSD (full)	P-value	PTSD (5 symptom)	P-value	PTSD (definition 1)	P-value	PTSD (2 cluster)	P-value	PTSD (majority)	P-value	PTSD (6 symptoms)	P-value
Gender												
*Female*	Ref	0.593	Ref	0.189	Ref		Ref		Ref		Ref	
*Male*	0.78		0.59		0.75	0.445	0.97	0.907	0.70	0.294	1.13	0.659
Age^	0.99	0.655	0.98	0.175	0.98	0.072	0.99	0.624	0.98	0.043*	0.999	0.959
Profession												
*Non clinical*	Ref	0.346	Ref	0.270	Ref		Ref		Ref		Ref	0.872
*Clinical*	0.67		1.11		0.90	0.765	1.23	0.477	0.75	0.377	0.95	
Depression												
*No*	Ref	<0.001*	Ref	0.001*	Ref		Ref		Ref		Ref	
*Yes*	4.89		3.92		4.31	<0.001*	7.41	<0.001*	6.56	<0.001*	18.93	<0.001*
Beirut Port Blast^	1.004	0.001*	1.004	0.001*	1.003	0.005*	1.004	<0.001*	1.004	<0.001*	1.004	<0.001*
Financial Score^	0.98	0.932	0.92	0.482	0.94	0.654	0.999	0.997	0.97	0.813	0.97	0.806
Covid-19 Score^	1.02	0.472	0.99	0.829	1.01	0.828	0.98	0.404	0.98	0.468	0.99	0.634
Prior Trauma Score^	2.06	<0.001*	1.74	0.001*	1.51	0.011*	2.04	<0.001*	1.81	<0.001*	3.24	<0.001*
Childhood Adversities												
*No CA*	Ref	<0.001*	Ref	<0.001*	Ref		Ref	<0.001*	Ref		Ref	<0.001*
*Any CA*	7.63		5.94		4.51	<0.001*	3.41		4.11	<0.001*	4.57	

*p-value < 0.05.

^^^continuous variable.

### 4.3 Multivariate

#### 4.3.1 Age

Age was not linked to PTSD at 6–7 months post-blast (**Table 6**), but at 2–2.5 years (**Table 7**), younger age predicted higher PTSD odds (full: OR = 0.95, p = 0.046; 5 symptoms: OR = 0.96, p = 0.029; definition 1: OR = 0.95, p = 0.007; majority: OR = 0.96, p = 0.009).

**Table 6:** Multivariable logistic regression of PTSD by definition type, 6–7 months post-Beirut port blast.

6–7 months post-blast	PTSD (full)		PTSD (5 symptom)		PTSD (definition 1)		PTSD (2 cluster)		PTSD (majority)		PTSD (6 symptom)	
	OR	p-value	OR	p-value	OR	p-value	OR	p-value	OR	p-value	OR	p-value
Gender												
*Female*	Ref	0.311	Ref	0.074	Ref	0.006*	Ref	0.104	Ref	0.109	Ref	0.226
*Male*	0.56		0.37		0.19		0.47		0.44		0.56	
Age^	0.996	0.844	1.01	0.775	1.02	0.234	1.01	0.605	1.02	0.411	1.02	0.327
Profession												
*Non clinical*	Ref	0.52	Ref	0.476	Ref	0.76	Ref	0.257	Ref	0.104	Ref	0.029*
*Clinical*	1.47		1.48		1.19		1.71		2.36		3.1	
Depression												
*No*	Ref	0.047*	Ref	0.064	Ref	0.020*	Ref	0.044*	Ref	0.018*	Ref	0.006*
*Yes*	3.91		3.5		5.38		5.09		6.81		11.17	
Beirut Port Blast^	1	0.022*	1	0.030*	1	0.087	1.004	0.031*	1.004	0.043*	1	0.083
Financial Score^	2.91	<0.001	2.64	<0.001*	3.02	<0.001*	1.92	<0.001*	3	<0.001*	2.51	<0.001*
Covid-19 Score^	1.02	0.678	1.05	0.223	1.11	0.015*	1.02	0.654	1.07	0.082	1.03	0.409
Prior Trauma Score^	1.29	0.299	1.43	0.133	1.53	0.082	1.52	0.048*	1.36	0.188	1.27	0.288
Childhood Adversities												
*No CA*	Ref		Ref		Ref		Ref		Ref		Ref	
*Any CA*	0.83	0.626	1.13	0.729	0.94	0.859	1.28	0.476	1.05	0.893	1.04	0.896

^^^continuous variable.

*p-value < 0.05.

**Table 7:** Multivariable logistic regression of PTSD by definition type, 2-2.5 years post-Beirut port blast.

2-2.5 years post-blast	PTSD (full)		PTSD (5 symptom)		PTSD (definition 1)		PTSD (2 cluster)		PTSD (majority)		PTSD (6 symptom)	
	OR	p-value	OR	p-value	OR	p-value	OR	p-value	OR	p-value	OR	p-value
Gender												
*Female*	Ref	0.125	Ref	0.021*	Ref	0.118	Ref	0.957	Ref	0.122	Ref	0.982
*Male*	0.29		0.21		0.42		1.02		0.47		1.01	
Age^	0.95	0.046*	0.96	0.029*	0.95	0.007*	0.993	0.607	0.96	0.009*	0.996	0.782
Profession												
*Non clinical*	Ref	0.174	Ref	0.89	Ref	0.471	Ref	0.781	Ref	0.227	Ref	0.827
*Clinical*	0.42		0.93		0.71		1.11		0.59		1.1	
Depression												
*No*	Ref	0.976	Ref	0.677	Ref	0.758	Ref	0.342	Ref	0.201	Ref	0.018*
*Yes*	0.98		0.76		1.21		1.89		2.12		5.62	
Beirut Port Blast^	1.01	0.218	1.004	0.043*	1.002	0.179	1.003	0.062	1.002	0.239	1	0.688
Financial Score^	0.92	0.698	0.85	0.34	0.92	0.6	1.07	0.577	1.02	0.872	1.07	0.611
Covid-19 Score^	1.07	0.152	0.998	0.951	1.03	0.43	0.97	0.216	1.01	0.856	0.98	0.409
Prior Trauma Score^	1.7	0.092	1.37	0.218	1.09	0.709	1.42	0.102	1.16	0.516	2.21	0.002*
Childhood Adversities												
*No CA*	Ref	<0.001*	Ref	<0.001*	Ref	<0.001*	Ref	0.004*	Ref	0.001*	Ref	
*Any CA*	3.56		3.21		2.71		2.32		2.4		2.04	0.008*

^^^continuous variable.

*p-value < 0.05.

#### 4.3.2 Depression

At 6–7 months, depression strongly increased PTSD odds across most definitions (full: OR = 3.91, p = 0.047; definition 1: OR = 5.38, p = 0.020; 2 cluster: OR = 5.09, p = 0.044; majority: OR = 6.81, p = 0.018; 6 symptoms: OR = 11.17, p = 0.006). At 2–2.5 years, depression was only significant for the six-symptom definition (OR = 5.62, p = 0.018).

#### 4.3.3 Beirut port blast

At 6–7 months post-blast, Beirut Port Blast exposure significantly predicted PTSD under multiple definitions (2 cluster: OR = 1.004, p = 0.031; majority: OR = 1.004, p = 0.043), but its effect weakened at 2–2.5 years.

#### 4.3.4 Finance

Financial difficulties were a robust predictor at 6–7 months across all PTSD outcomes (ORs 1.92–3.02; all p < 0.001) but not at 2–2.5 years.

#### 4.3.5 Covid-19

COVID-19 stressors were largely non-significant in both waves, except for one PTSD definition (definition 1) at 6–7 months (OR = 1.11, p = 0.015).

#### 4.3.6 Prior trauma

Prior trauma history showed inconsistent effects—significant at 6–7 months post-blast for two-cluster PTSD (OR = 1.52, p = 0.048) and at 2–2.5 years post-blast for six-symptom PTSD (OR = 2.21, p = 0.002).

#### 4.3.7 ACE

The most striking 2–2.5-year post-blast finding was the pronounced role of childhood adversities, a highly significant predictor across all PTSD outcomes (full: OR = 3.56, p < 0.001; 5 symptoms: OR = 3.21, p < 0.001; definition 1: OR = 2.71, p < 0.001; 2 cluster: OR = 2.32, p = 0.004; majority: OR = 2.40, p = 0.001; 6 symptoms: OR = 2.04, p = 0.008), in contrast to its non-significance at 6–7 months post-blast.

## 5 Discussion

Exploring long-term PTSD predictors is key for clinical and public health action. In just five years, the Lebanese population has faced multiple traumas—COVID-19, a financial crisis, and the Beirut Port explosion, whose destruction resembled that of a nuclear blast. The literature has long established the significant psychological burden and functional impairment such traumatic events can incur (3, 4), even in the absence of a full PTSD diagnosis.

This study marks the first of its kind to explore not only multiple predictors of full-threshold PTSD but all six validated definitions of subthreshold PTSD (5) with the aim of advancing the understanding of trauma psychopathology in a longitudinal cohort of Lebanese HCWs exposed to multiple traumatic events in the span of five years.

The results revealed ACEs as the strongest predictor of PTSD symptoms showcasing significant association with both full-threshold PTSD and all definitions of subthreshold PTSD in 2-2.5 years post-blast; a notable distinction from findings in 6–7 months post-blast, where ACEs were not significant predictors whereas acute events such as Lebanon’s financial crisis, the Beirut Port Blast, and COVID-19 exposure were significantly predictive of PTSD outcomes. This weaker association may indicate a temporal decline of the identified predictors, namely a diminishing predictive power of acute stressors. The declining predictive role of one such acute stressor, financial hardship, can be explained by the widespread dollarization in the years following the national economic collapse, which may have stabilized inflation short-term, as well as the dissociation from the failing banking system and reliance on alternative models like informal financial practices (38).

This underscores the need for longitudinal assessments to better understand the evolution of PTSD predictors over time despite the documented impact of acute stressors within the literature (35, 39).

The emergence of ACEs as predictors and the fading role of acute traumas 2.5 years post-event demonstrates the temporal dynamism of trauma vulnerability. Large-scale cross-sectional data by Kessler et al. (40) established the delayed predictive role of ACEs, linking childhood abuse, neglect, maladaptive family functioning, and parental mental illness to adult psychopathology, especially PTSD. Notably, ACEs remained the only consistent predictor of both subthreshold and full PTSD years after acute polytrauma.

While the hypothesis posited that proximal stressors (Beirut Blast, COVID-19, financial collapse) would be stronger predictors given their immediacy and intensity, our findings revealed distal stressors as most predictive. As acute stress responses wane, the deeper-rooted impacts of ACEs become more prominent, shaping PTSD symptom trajectories. This interpretation is supported by multilevel modeling studies showing that individuals with enduring PTSD symptoms are more likely to have a history of childhood trauma (41).

### 5.1 Developmental vulnerability and long-term impact of ACEs

A transition from reactivity to acute traumatic events to a developmental vulnerability can be posited in which the work of Berthail et al. (42) establishes the role of early adversity in undermining emotional regulation, cognitive reappraisal, and stress recovering mechanisms implicated in psychological well-being. This is further explained by neurobiological studies showcasing deficits, both functional and structural, at the levels of the hippocampus and prefrontal cortical regions, necessary for post-trauma recovery and implicated in contextual procession and emotional regulation explaining decreased adaptation and PTSD symptom persistence (41–43). It can then be inferred, that individuals with fewer or no ACEs may experience better recovery trajectories, benefiting from more intact regulatory networks and adaptive cognitive frameworks.

An alternative interpretation of these findings is that the enduring effects of ACEs reflect survival-oriented responses to chronically threatening developmental environments. Patterns of emotional regulation, defensive functioning, and coping may emerge as means of managing sustained adversity, even if they may later hinder recovery following subsequent trauma exposure; empirical support for this developmental pathway links exposure to ACEs with PTSD and disturbances in self-organization symptoms through immature defense mechanisms and maladaptive coping processes in adolescents particularly, highlighting how prolonged adversity may shape enduring psychological response patterns (44). This perspective is consistent with research on chronic trauma and Complex PTSD (CPTSD), which conceptualizes prolonged interpersonal adversity as shaping not only core PTSD symptoms but also disturbances in self-organization, including affective dysregulation, negative self-concept, and relational difficulties (45). Future studies using CPTSD-focused measures, such as the International Trauma Questionnaire developed by Cloître et al. (46), may therefore help clarify whether the persistent predictive effect of ACEs in polytraumatized populations reflects broader posttraumatic adaptations extending beyond conventional PTSD symptom frameworks (47–49).

The diminished explanatory power of acute predictors years post-trauma, unlike ACEs that emerge as dominant determinants of chronic PTSD, may be explained by research on first responders, consistent with our sample. Given the repeated exposure to trauma, these individuals may develop functional suppression abilities which serve as coping mechanisms in high-stress contexts (43, 50). The aforementioned is further established by Haim-Nachum et al. (51), whose findings highlight the critical predictive role of ACEs following trauma exposure in adulthood as opposed to adolescent or adult adversities. This underscores the need for trauma-informed approaches that consider both developmental timing and chronicity in PTSD progression.

### 5.2 Trauma type and timing

The isolated association of the Beirut Blast, a human-made disaster, with only the “Five Symptoms” subthreshold definition 2.5 years later further underscores the importance of trauma type, proximity, and injury status. This definition requires the presence of at least one symptom from each PTSD cluster but does not impose the higher symptom counts required by other definitions. As such, it may function as a particularly sensitive indicator of low-grade but widely distributed trauma-related distress. Over time, acute trauma responses often attenuate, with many individuals no longer meeting full or cluster-based diagnostic thresholds. However, residual symptoms may persist across multiple domains without reaching the severity required by stricter definitions. The “Five Symptoms” framework may therefore capture subclinical, lingering manifestations of trauma exposure, reflecting a residual psychological footprint of the event rather than an active disorder. This interpretation aligns with longitudinal trauma research suggesting that the long-term impact of mass disasters may manifest less as full PTSD and more as persistent, low-intensity symptom constellations that remain detectable only through lower-threshold screening criteria (35, 39).

Another explanation, informed by Diamond et al. (52), offers insights into why PTSD symptoms linked to COVID-19, and the financial crisis diminished by 2-2.5 years post-blast, unlike symptoms associated with ACEs. The distinction is that intentional trauma (e.g., abuse, neglect) is more likely to be linked to chronic PTSD than non-intentional trauma (e.g., COVID-19, financial crisis), owing to its interpersonal nature, betrayal of trust, and disruption of core beliefs. In contrast, non-intentional traumas may permit external attribution and adaptive coping, making remission more likely. This distinction explains the enduring impact of ACEs, whose developmental timing and relational nature influence recovery long after more recent traumas. Ford and Seedat (53) propose a plausible hypothesis for why COVID-19 did not emerge as a significant PTSD predictor, which may lie in its psychological impact. Specific stressors (death of loved ones, confinement, social isolation, resource deprivation) have indeed been linked to PTSD symptoms. This distinction suggests that generalized COVID-19 exposure may lack the traumatic specificity associated with PTSD without the stressors established within the literature. Without individualized assessments of these elements, COVID-19 may not present as a salient predictor of PTSD in polytrauma models. This lens, showing the predictive role of ACEs in 2-2.5 years post-blast through trauma types, aligns with the work of Dougall et al. (54) on dissimilar vs. similar trauma and stress sensitization in frontliners —perceived threat from recent trauma predicts initial symptoms, but long-term outcomes are better explained by earlier, dissimilar traumas, particularly childhood adversity. This supports the sensitization hypothesis proposed by their study (54), where early trauma different in nature from more recent healthcare traumas (e.g., COVID-19, Beirut Blast), primes stress-response systems, increasing vulnerability to later distress.

### 5.3 Diagnostic overlap between depression and PTSD

Another important dimension involves the predictive specificity of depression, which was only associated with the “Six-Plus” subthreshold definition capturing symptom burden in 2-2.5 years post-blast. This finding draws attention to the substantial symptom overlap between depression and PTSD, particularly regarding emotional numbing, anhedonia, and concentration difficulties (1, 55). Recent work by Al Barathie & Karam (56) informs this hypothesis, identifying a two-factor PCL-5 structure in a cumulative trauma-exposed Lebanese sample, with one factor mapping trauma-specific symptoms and the other reflecting general distress aligned with depressive and anxiety features.

Taken together, the link between depression and the “Six-Plus” definition likely reflects overlapping, non-specific emotional dysregulation rather than discrete trauma pathology. This finding highlights the diagnostic imprecision in current PTSD frameworks, particularly in populations with chronic, overlapping stressors, strengthening the case for transdiagnostic models of assessment and intervention to better inform clinical and research efforts.

## 6 Limitations

Several limitations should be acknowledged. First, the study was conducted among healthcare workers from a single tertiary hospital in Beirut and the sample were predominantly females, which may limit the generalizability of the findings to other settings or populations. Second, although validated screening tools such as the PCL-5 and PHQ-9 were used, several variables, including adverse childhood experiences and exposure-related stressors, were assessed through self-report and may therefore be subject to recall or reporting bias. Third, participation was voluntary, which may introduce selection bias. Moreover, the analyses were conducted only among participants who have complete data in waves 3& 4, and we did not perform a formal attrition analysis. Yet, it is important to note that a substantial number of healthcare workers were lost to follow-up due to external factors, primarily migration for financial reasons. While this reduces the likelihood of “healthy worker” bias, we cannot fully exclude the possibility that those lost to follow-up may have differed in ways that could affect PTSD outcomes. Moreover, only three childhood adversities documented in the literature were evaluated in this study. Finally, PTSD outcomes were measured using a screening instrument rather than clinician-administered diagnostic interviews. Future research should replicate these findings in larger multicenter samples, using diagnostic interviews for PTSD and adopting more inclusive instruments, such as the full ACEs questionnaire. Additionally, there should be a further examination of the mechanisms linking childhood adversity to persistent PTSD symptoms and exploration of trauma-informed interventions for healthcare workers exposed to repeated large-scale stressors.

## Data Availability

The raw data supporting the conclusions of this article will be made available by the authors, without undue reservation.

## Glossary

ACE(s): Adverse Childhood Experience(s)
CA(s): Childhood Adversity(ies)
CI: Confidence Interval
COVID-19: Coronavirus Disease 2019
DSM-5: *Diagnostic and Statistical Manual of Mental Disorders*, Fifth Edition
HCW(s): Healthcare Worker(s)
IDRAAC: Institute for Development, Research, Advocacy, and Applied Care
OHRP: Office for Human Research Protections
OR: Odds Ratio
PCL-5: *Posttraumatic Stress Disorder Checklist for DSM-5*
PHQ-9: *Patient Health Questionnaire-9*
PTSD: Posttraumatic Stress Disorder
SD: Standard Deviation
SGHUMC: Saint George Hospital University Medical Center
