# Supplementary material for: Unmasking deep-rooted trauma: long-term effects of childhood adversities on posttraumatic stress disorder in healthcare workers facing acute multi-trauma
Source: Front Psychiatry. 2026 Apr 14;17:1788332. doi: 10.3389/fpsyt.2026.1788332 (PMC13121125; doi:10.3389/fpsyt.2026.1788332)
Supplement: Supplementary file 1 [file Table1.docx]

Table S1: Beirut Port Blast Exposure

|  | **Beirut Blast Weighted Score Questions** | **Weight** |
| --- | --- | --- |
| **1** | Were you physically injured? | No=0  Mildly=40  Moderately=60  Severely=90 |
| **2** | If you were physically injured, did you have trouble getting the needed medical attention? | No=0  Yes + Mildly Injured=40  Yes + Moderately Injured=70  Yes + Severely Injured=90 |
| **3** | Did any of your loved ones die? | No=0  Yes = 99.5 |
| **4** | Were any of your loved ones severely injured (but did not pass away)? | No=0  Yes=80 |
| **5** | Were any of your loved ones injured moderately or mildly? | No=0  Yes=60 |
| **6** | Was the place where you live affected by the explosion? | No=0  Moderately=47.5  Severely=70  Completely=85 |
| **7** | Did you participate in rescue efforts of patients in the hospital or other injured people outside the hospital? | No=0  Yes, light injuries=50  Yes, moderate injuries=60  Yes, severe injuries=80 |
| **8** | Did you see any mutilated / dead bodies? | No=0  Yes=80 |

Table S2: Items and scoring of the childhood adversity weighted score

|  | **Childhood Adversities Weighted Score Questions** | **Weight** |
| --- | --- | --- |
| **1** | Did you experience neglect during childhood? | No=0  Yes=1 |
| **2** | Did you experience physical abuse during childhood? | No=0  Yes=1 |
| **3** | **Did you experience sexual abuse during childhood?** | No=0  Yes=1 |

Table S3: COVID-19 Exposure Score

|  | **COVID-19 Weighted Score Questions** | **Weight** |
| --- | --- | --- |
| **1** | I have experienced violence due to being a health worker during the pandemic. | No=0  Yes=5 |
| **2** | During the past week, have you been close to patients who were suspected or confirmed cases of COVID-19? | No=0  Yes=3 |
| **3** | Has the personal protective equipment you used been provided by your institution (your principal place of work)? | No=0  Yes=2 |
| **4** | Have any of your loved ones passed away due to COVID-19? | No=0  Yes=5 |
| **5** | How many days have you been in isolation for being a suspected or confirmed case of COVID-19, since the beginning of the pandemic? | No=0  Yes=4 |
| **6** | Since the beginning of the pandemic, have any of the patients with COVID-19 that you directly cared for passed away? | No=0  Yes=5 |
| **7** | Have you felt stigmatized or discriminated against as a health worker due to the COVID-19 pandemic? | No=0  Yes=4 |
| **8** | Since the beginning of the pandemic, have you had to decide how to prioritize patients with COVID-19? | No=0  Yes=5 |
| **9** | How worried are you about getting COVID-19? | No=0  Yes=4 |
| **10** | How worried are you about infecting your loved ones with COVID-19? | No=0  Yes=4 |
| **11** | To what extent do you trust that your workplace can manage the COVID-19 pandemic? | No=0  Yes=3 |
| **12** | To what extent do you trust that your government can manage the COVID-19 pandemic? | No=0  Yes=1 |
| **13** | Have you received specific training on how to prevent transmission of COVID-19? | No=0  Yes=1 |
